# Human Hepatic HepaRG Cells Maintain an Organotypic Phenotype with High Intrinsic CYP450 Activity/Metabolism and Significantly Outperform Standard HepG2/C3A Cells for Pharmaceutical and Therapeutic Applications

**DOI:** 10.1111/bcpt.12631

**Published:** 2016-07-15

**Authors:** Leonard J. Nelson, Katie Morgan, Philipp Treskes, Kay Samuel, Catherine J. Henderson, Claire LeBled, Natalie Homer, M. Helen Grant, Peter C. Hayes, John N. Plevris

**Affiliations:** ^1^ Hepatology LaboratoryRoyal Infirmary of EdinburghUniversity of EdinburghEdinburghUK; ^2^ Scottish National Blood Transfusion ServiceResearch Development and Innovation DirectorateCell Therapy GroupEdinburghUK; ^3^ Department of Biomedical EngineeringUniversity of StrathclydeGlasgowUK; ^4^ Mass Spectrometry Core LaboratoryWellcome Trust Clinical Research FacilityQueen's Medical Research InstituteEdinburghUK

## Abstract

Conventional *in vitro* human hepatic models for drug testing are based on the use of standard cell lines derived from hepatomas or primary human hepatocytes (PHHs). Limited availability, interdonor functional variability and early phenotypic alterations in PHHs restrict their use, whilst standard cell lines such as HepG2 lack a substantial and variable set of liver‐specific functions such as CYP450 activity. Alternatives include the HepG2‐derivative C3A cells selected as a more differentiated and metabolically active hepatic phenotype. Human HepaRG cells are an alternative organotypic co‐culture model of hepatocytes and cholangiocytes reported to maintain *in vivo*‐like liver‐specific functions, including intact Phase I–III drug metabolism. In this study, we compared C3A and human HepaRG cells using phenotypic profiling, CYP450 activity and drug metabolism parameters to assess their value as hepatic models for pre‐clinical drug testing or therapeutics. Compared with C3As, HepaRG co‐cultures exhibit a more organotypic phenotype, including evidence of hepatic polarity with the strong expression of CYP3A4, the major isoform involved in the metabolism of over 60% of marketed drugs. Significantly greater CYP450 activity and expression of CYP1A2, CYP2E1 and CYP3A4 genes in HepaRG cells (comparable with that of human liver tissue) was demonstrated. Moreover, HepaRG cells also preferentially expressed the hepatic integrin α_5_β_1_ – an important modulator of cell behaviour including growth and survival, differentiation and polarity. Drug metabolite profiling of phenacetin (CYP1A2) and testosterone (CYP3A4) using LC‐MS/MS and HPLC, respectively, revealed that HepaRGs had more intact (Phase I–II) metabolism profile. Thus, HepaRG cells significantly outperform C3A cells for the potential pharmaceutical and therapeutic applications.

Withdrawal of licensed drugs from the market despite passing both pre‐clinical *in vitro* and *in vivo* toxicity testing continues to be a significant problem. Development of improved *in vitro* human hepatic models for pre‐clinical drug testing is required to more closely resemble the *in vivo* hepatic environment and minimize interspecies differences 1. Hence, *in vitro* toxicological models based on human hepatic cell lines would provide more comparable and informative readout than animal models. In order to develop a physiologically relevant, sustainable and reproducible *in vitro* culture system, the choice of a suitable cell line is critical. Human hepatoma‐derived cell lines such as HepG2 and Huh7 are commonly used in early drug safety assessment 2, but often fail to predict hepatotoxic drugs. The ideal cell model should maintain metabolic pathways such as key CYP450 enzyme activities, possess an intact drug transporter system (hepatic polarity) and be able to offer mechanistic insight into the effects of drugs at both the cellular and molecular level. In addition, such culture systems should have a stable metabolic phenotype to further ensure reproducibility and flexibility of use. Primary human hepatocytes (PHHs) are the preferred *in vitro* model for many pharmaceutical and therapeutic approaches. However, limited availability, interdonor functional and genetic variability and early phenotypic alterations of PHH cultures restrict their application, such as repeat/chronic toxicity studies, or availability for cell therapeutics. Indeed, the integrity of PHHs used for modelling metabolic processes may be in question; given the inherent phenotypic instability of PHHs since upon isolation, the cells are in a state of pre‐apoptotic stress with the differences in stability of individual CYP450s in culture 3. This represents a major challenge for pharma where the standardization and development of more practical, sustainable and stable physiologically relevant alternatives are prerequisite for improving pre‐clinical testing outcome.

Although hepatoblastoma‐derived cancer cells, such as the HepG2 cell line, are an inexpensive and convenient model, widely used in pre‐clinical drug testing, they lack a substantial set of liver‐specific functions, particularly CYP450 activity 4. The HepG2/C3A cell line (herein designated C3A cells) is a clonal derivative of the HepG2 cell line. C3A cells were selected as a more differentiated and metabolically active hepatic phenotype, compared with the parent HepG2 cell line 5.

Previously, we demonstrated the enhancement of the C3A cell phenotype, including CYP3A4 activity/albumin synthesis, by co‐culture with human endothelial cells 5, and through ‘metabolic’ preconditioning 6, whilst others have used tissue engineering approaches to augment C3A cell metabolism 7, 8. Forced transfection of HepG2 hepatic cell lines with CYP2E1‐containing plasmids is also performed for highly specific applications, such as ethanol toxicity studies 9. Notably, C3A and human HepaRG hepatic cell lines have been implemented as the biological component of bioartificial liver systems (BALs) 10, suggesting that both cell types possess a sufficiently ‘organotypic’ phenotypic and functional properties to support patients with acute liver failure. However, it is not known whether conventional C3A cell monocultures are a practicable model for use in metabolic studies for pre‐clinical drug testing or for clinical applications such as BALs 11. Indeed, limited functionality, low CYP activity or poor sustainability would represent significant barriers, for BAL application 12, 13.

The human HepaRG hepatic cell line has emerged as a potential surrogate to PHHs for pre‐clinical hepatotoxicity assays 14. HepaRGs are a unique (intrinsic), terminally differentiated co‐culture of hepatocyte‐ and cholangiocyte‐like cells containing many functional and phenotypic similarities with PHH 15. These cells were procured from a young adult female with hepatocarcinoma 16. HepaRGs are a highly reproducible cell line, without the donor variability seen in PHHs, and as such ensure a consistent and phenotypically stable cell line, presenting a potentially more standardized model 17. HepaRG cells retain some of the major CYP450 pathways and Phase II enzymes as well as the production of glucose/glycogen and urea 15, 16, 17. These cells also show functional polarity, a hallmark of *in vivo* hepatocyte organization 15, with intact Phase III drug transporters. These properties are generally not evident in ‘standard’ human hepatic cell lines monocultures. Given their potential for pharmaceutical applications and cell therapeutics including BALs, studies comparing such widely used human hepatic cell lines are surprisingly limited, whilst direct comparisons between C3A cells and HepaRG cells have not been previously reported. In this study, we aimed to compare the phenotypic and metabolic parameters, including CYP450 activity and metabolism between HepG2/C3A and human HepaRG cells, to assess their value as suitable hepatic models for pre‐clinical drug testing and therapeutics.

## Materials and Methods

### Cell culture

C3A cells (HepG2/C3A), derivative of HepG2: ATCC^®^ CRL‐10741^™^, were cultured on Corning plates in minimum essential medium Eagle (MEME+; Sigma Aldrich) with 10% foetal bovine serum (FBS, Life Technologies, Paisley, UK) and 1% penicillin/streptomycin (Life Technologies). The culture was kept at 37°C 5% CO_2_ until ~80% confluency. HepaRG cells (HRG116 terminally differentiated cells, BioPredic international, Rennes, France) were seeded (following the suppliers protocols) on Corning plates and cultured to confluence at 37°C 5% CO_2_ for 7 days.

### Immunocytochemistry

On culture day 8, the cells were fixed in 4% paraformaldehyde (Sigma Aldrich), permeabilized with 0.1% Triton X‐100 in tris‐buffered saline (TBS), then blocked with TBS containing 5% bovine serum albumin (BSA; Sigma Aldrich) before incubation with primary (rabbit) anti‐human CYP3A4 antibody (AB1254, Chemicon, Millipore, Hertfordshire, UK) (1:800) and TRITC‐conjugated phalloidin (Sigma Aldrich) (1:100). The cells were then treated with appropriate secondary anti‐rabbit antibody (Alexa Fluor^®^ 488) prior to (nuclear) staining with DAPI (1:5000) for 5 min. Cell morphology was assessed under phase contrast and for immunocytofluorescent staining using an EVOS AUTO FL microscope (Life Technologies). Images were merged using ImageJ 1.47v (National Institute of Health, Bethesda, MD, USA).

### Biotransformation potential

#### Cytochrome P450 enzyme activity

Cells were treated for 24 hr with prototypical inducers 50 μM omeprazole (CYP1A2) or rifampicin (CYP3A4), with or without the addition of CYP450 isoform‐specific inhibitors 25 μM fluvoxamine (CYP1A2) or ketoconazole (CYP3A4), in MEME (C3As) or hepatocyte induction medium (HepaRGs). The cells were then washed twice with HBSS. Specific CYP450 enzyme activity was subsequently assessed using a non‐lytic luminescence assay using specific kits for CYP1A2 and CYP3A4, following the manufacturer's instructions (P450‐Glo, Promega, Southampton, UK). Bioluminescent signals were detected with a GloMax‐Multi+ Microplate Multimode Reader (Promega). Individual luminescent assay readings were background‐corrected and normalized to cellular ATP content.

#### Phase I and II metabolism of phenacetin and testosterone

In order to profile the relative drug metabolism in each cell type, metabolites formed by CYP1A2 or CYP3A4 hepatic metabolism were assessed after the exposure to 50 μM phenacetin (CYP1A2), or testosterone (CYP3A4), for 2 hr at 37°C. Reactions were stopped on ice, and cell supernatant (medium) samples were stored at −80°C before analytical measurements using HPLC (testosterone) or LC‐MS/MS (phenacetin).

#### Phenacetin metabolism: Liquid chromatography–mass spectrometry

Phenacetin metabolism was assessed using an ABI Applied Biosystems 5500 QTrap Mass spectrometer and AB Sciex software, Analyst 1.5.1, and LightSight software to identify the metabolites. Methods are described in detail in Supporting Information.

#### Testosterone

Relative turnover and metabolic breakdown of testosterone was assessed by HPLC‐UV with absorbance at 254 nm. Samples were enriched with an internal standard (20 μg/ml 11 α‐hydroxyprogesterone in methanol) and extracted with dichloromethane before they were resuspended in 30% methanol and injected (25 μl) onto an Eclipse XD8‐C18 3 μm 3 × 100 mm column (Agilent, Cheadle, UK). A series of calibration standards were prepared containing 6β, 7α, 16α, 16β and 2α‐hydroxytestosterone, plus internal standard. Androstenedione and testosterone were analysed at the start of each run at 0.25, 0.5, 1, 2.5, 5, 7.5 and 10 nmoles in 30% methanol.

#### Flow cytometry

Integrin expression of C3A cells was compared with HepaRG cells, including the progenitor HepaRG101 cell line, and after the differentiation to terminally differentiated HepaRG116 cells. This allows the assessment of integrin expression in C3A cells, known to express foetal markers (e.g. alpha‐fetoprotein), with both the HepaRG101 (hepatoblast‐like) progenitors and their derivatives (HepaRG116), as comparators. To assess the expression of integrins, single cell suspensions were enzymatically recovered using xeno‐free Tryple (Life Technologies) – which protects cellular surface proteins – washed and resuspended in FACS–PBS (PBS containing 0.1% BSA and 0.1% sodium azide), for simultaneous staining with a panel of fluorochrome‐labelled integrin antibodies: CD29‐BV510, CD49f‐BV451, CD49d‐FITC, CD49c‐PE, CD49a‐APC‐Vio770, CD49b‐PE‐Vio770, CD49e‐APC (all from Miltenyi Biotec). The cells were incubated with antibodies at 4°C for 20 min., washed twice and resuspended in FACS–PBS. Unstained cells were included as controls, whilst dead cells and debris were excluded from the analysis based on scatter characteristics. Data for at least 10,000 live events per sample were acquired using a MACSQuant Analyzer (Miltenyi Biotec) and were analysed using FlowJo version 9.6.7 software (FlowJo LLC). Data are presented as percentage positive staining.

### Statistical analysis

All experiments were performed with three to eight technical replicates from a minimum of three independent biological experiments. Results are presented as mean ± standard error of the mean (S.E.M.), and individual groups were (generally) compared with a two‐tailed unpaired Student's *t*‐test to test significance as indicated. A *p*‐value lower than 0.05 was considered statistically significant.

## Results

### Phenotypic profiling of HepaRG and HepG2/C3A cells

**Figure 1:**
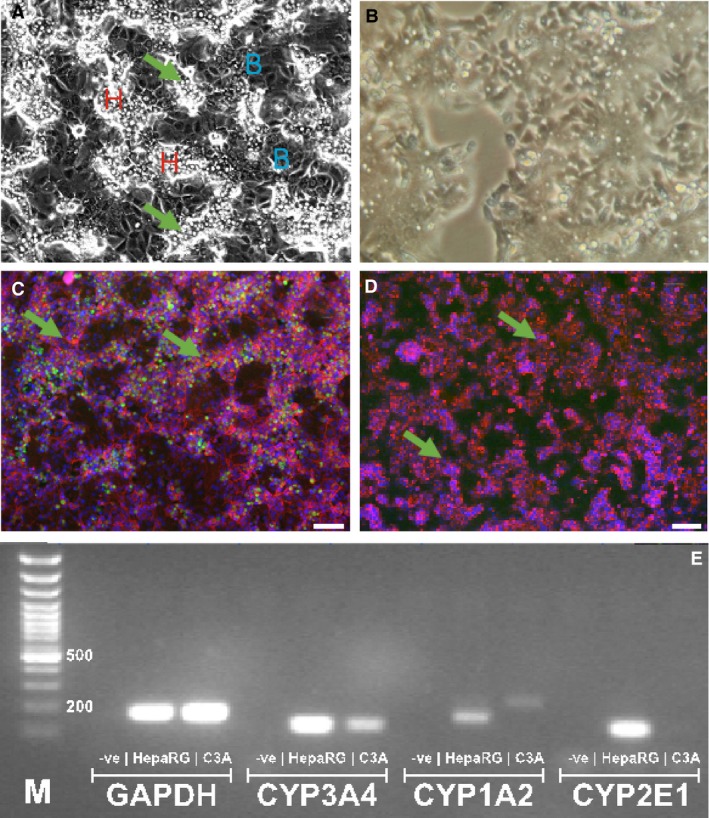
Phenotypic profiling of human hepatic HepaRG compared with C3A cells HepaRG cells are an intrinsic co‐culture of hepatocyte‐ and cholangiocyte (biliary)‐like cells (denoted *B*, in panel A) with islands of hepatocytes (*H*) with sharp refractile borders/round nuclei, and bile canaliculi (arrows) interspersed with a thin layer of cholangiocytes (panel A). C3As form a confluent monolayer of hepatocyte‐like cells, with less prominent bile canaliculi/refractile borders (B). (C) HepaRG cells exhibit a highly differentiated phenotype (culture day 8): tri‐colour fluorescent staining revealed extensive hepatic CYP3A4 enzyme activity (green); punctate staining of F‐actin bands, indicative of bile canalicular structures [red phalloidin staining, green arrows], with *in vivo*‐like hepatic cords and cholangiocyte‐like cell ‘voids’. In contrast, in C3A cells CYP3A4 enzyme activity is undetected (D), with low CYP450 gene expression/metabolic competence in comparison with high CYP2E1, CYP1A2 and CYP3A4 gene expression in HepaRG cells, after end‐point PCR (E).

HepaRG cells formed terminally differentiated *in vivo*‐like hepatic cords and cholangiocyte‐like cells (fig. 1A) with functional polarity, as demonstrated by punctate staining of F‐actin bands indicative of bile canalicular structures (phalloidin staining, fig. 1C); these could also be observed in C3A cells (fig. 1D), although with less pronounced staining. In contrast, high CYP3A4 protein expression (fig. 1C; green staining) with strong expression of CYP2E1, CYP1A2 and CYP3A4 genes was evident only in HepaRGs, indicative of high metabolic competence for drug metabolism (fig. 1E).

### Biotransformation potential

#### CYP450 enzyme activity and specificity

**Figure 2:**
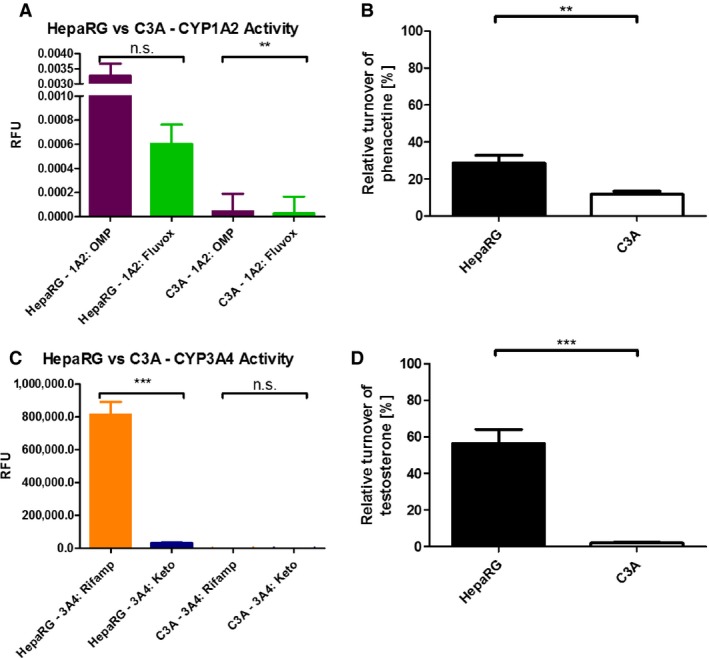
Metabolic profiling of human hepatic HepaRG compared with C3A cells. Measurement of prototypical inducers omeprazole (CYP1A2) and rifampicin (CYP3A4) in both HepaRG and C3A cells (A, C). Fluvoxamine, a highly specific inhibitor of CYP1A2, and ketoconazole, a highly specific inhibitor of CYP3A4, were used to demonstrate the specificity. Relative metabolic turnover (i.e. of total drug added to culture medium) of phenacetin (B) and testosterone (D). Results are presented as mean ± S.E.M., and individual groups were compared with a two‐tailed, unpaired Student's *t*‐test to test the significance as indicated. A *p*‐value <0.05 was considered statistically significant. *p* < 0.01**; *p* < 0.001***.

HepaRG CYP1A2 (fig. 2A) and CYP3A4 (fig. 2C) activities were significantly higher than those measured in HepG2/C3A cells [*p* < 0.0001]. In fact, relative luminescence measured in HepG2/C3A cells was at levels of blank controls. Specificity of CYP450 isoform induction was confirmed with the specific inhibitors: fluvoxamine (CYP1A2) and ketoconazole (CYP3A4).

#### Phase I and II metabolism of phenacetin and testosterone

To further investigate the extent of CYP1A2 and 3A4 metabolic activity in each cell type, phenacetin (CYP1A2) metabolism and testosterone (CYP3A4) metabolism were used to assess the metabolic competency. Compound turnover, and the ratio of analyte to metabolite, was assessed using LC:MS/MS (phenacetin) and HPLC (testosterone). Compared with C3A cells, HepaRG cells showed significantly higher *turnover* of phenacetin (28.5 ± 4.4% *versus* 11.7 ± 0.6%; *p* < 0.01) and testosterone (56.5 ± 7.6% *versus* 2.0 ± 0.2%; *p* < 0.001, respectively), with a wider spectrum and more refined metabolic breakdown of phenacetin and testosterone in HepaRG cells.

#### Phenacetin

**Figure 3:**
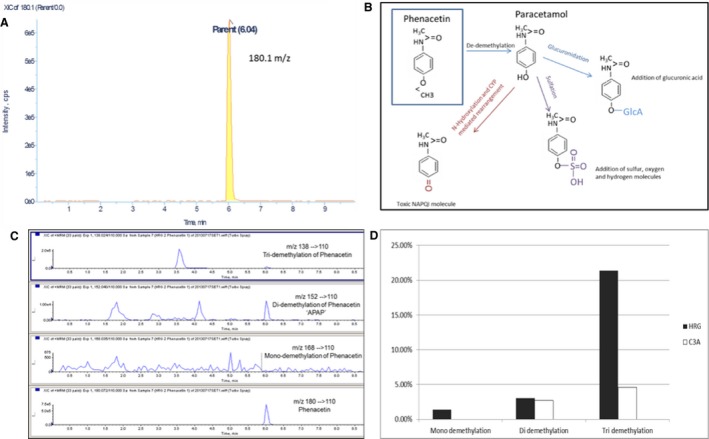
Representative chromatograph extracted from Light Sight software: Peak of parent compound phenacetin at its unique 180.0 m/z ratio (A). Schematic diagram showing Phase I and Phase II metabolism of phenacetin: Phase I (de‐demethylation) of phenacetin to paracetamol (acetaminophen), and Phase II secondary metabolites of acetaminophen via the processes of glucuronidation, sulphonation and N‐hydroxylation (B). LC‐MS chromatograms: Representative chromatograms of mono‐, di‐ and tri‐demethylation of HepaRG cells after challenge with phenacetin. The parent compound phenacetin is shown at 180 m/z, mono‐demethylation shown at 168 m/z, di‐demethylation 152 m/z and tri‐demethylation 138 m/z (C). (D) Comparison of mean % turnover of demethylation (comparing mean % of mono‐, di‐ and tri‐demethylation) between HepaRG and C3A cells.

HepaRG cells yielded a higher turnover of phenacetin and gave a clear yield ratio of analyte to metabolite via end‐point analysis (fig. 2B). We were able to characterize de‐demethylation of our substrate, phenacetin, by observing the significant mono‐ and tri‐demethylation of the parent metabolite into paracetamol (fig. 3B–D). In addition, characteristic secondary metabolites of sulphonation and glucuronidation were detected (data not shown). Such broad‐spectrum metabolism is not seen in C3A cells, where no secondary metabolites were detected.

#### Testosterone

**Figure 4:**
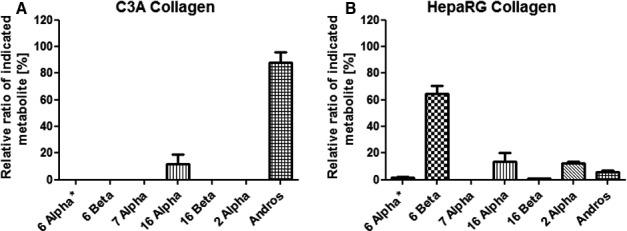
Drug metabolite profiling of testosterone (CYP3A4) using HPLC. Metabolism of testosterone in HepaRG cells on culture day 8 (A) showed a significant level of the major secondary metabolite 6‐beta‐hydroxytestosterone and a wider range of secondary metabolites compared with in C3A cells (B).

Given that each compound has its own unique absorbance, the chromatographic elution time of testosterone was compared in *in vitro* samples with that of known calibration standards, and HPLC‐UV absorbance was assessed using a photodiode array detector. In contrast with C3As, HepaRG cells metabolized >50% of (total) testosterone, producing a major metabolite (6‐β‐hydroxytestosterone), as well as a panel of other secondary metabolites (fig. 4A), whilst C3A cells only showed the presence of relatively minor metabolites, with low turnover of the parent compound (fig. 4B). Using HPLC, the relative *turnover* of testosterone by hydroxylation (see fig. 3D) was determined in HepaRG cells, whilst no trace of this metabolite is present in C3A cells.

#### Flow cytometric integrin expression in HepaRG and C3A cells

**Figure 5:**
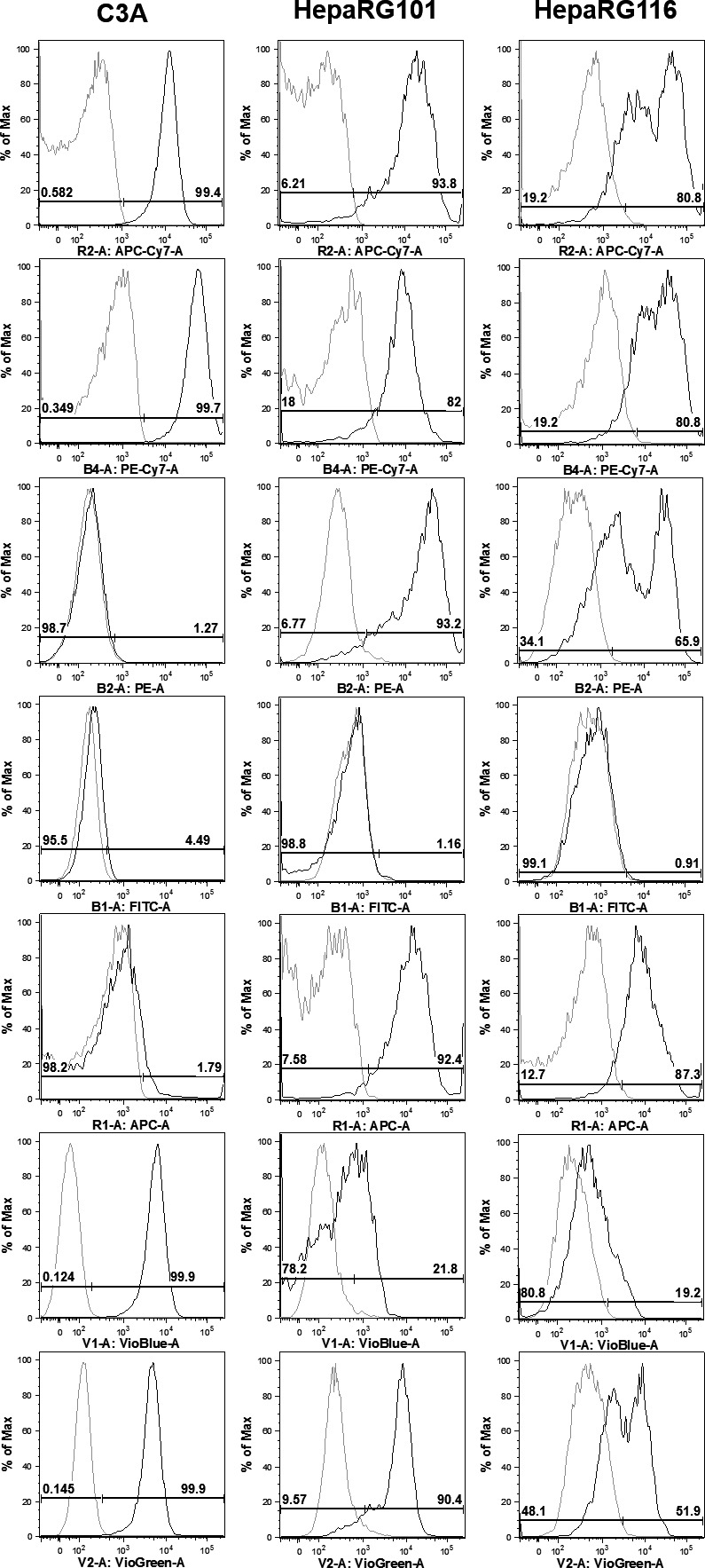
Flow cytometry profiling of integrin expression in HepaRG and C3A cells. Flow cytometry histograms comparing integrin expression (CD49a‐f: α1–α6) and CD29 (integrin β1) between C3A, HepaRG101 and HepaRG116 cell lines (dark lines), and isotype controls (grey lines). CD49d was not expressed by any of the three lines. The expression of CD49a, CD49b and CD29 was comparable between all 3 cell lines, although a proportion of HepaRG116 cells had down‐regulated expression. Whereas the expression of CD49e by HepaRG101 cells was maintained on HepaRG116 cells, but absent from C3A cells. This was also true for the expression of CD49c, except that there was some down‐regulation of expression by HepaRG116 cells. Conversely, C3A expressed CD49f, whilst it was absent or low on HepaRG101 and 116 cells.

Expression of integrins CD49a‐f (α subunits 1–6) and CD29 (β_1_ subunit) by C3A, HepaRG101 and HepaRG116 cells is shown in fig. 5. C3A cells expressed CD49a, CD49b, CD49f and CD29, but showed no staining for CD49c‐e. The immature/undifferentiated hepatoblast‐like cell line HepaRG101 showed a comparable phenotype, expressing CD49a, CD49b, CD49f, although the frequency and level of expression of CD49f was lower than that for C3A. In addition, HepaRG101 cells expressed CD49e, which was absent from C3A cells. HepaRG116 differentiated cells show hepatocyte, biliary and epithelial cells morphologically (fig. 5).

## Discussion

Appropriate *in vitro* human models are urgently required to help reduce high drug attrition rates by detecting toxicity earlier and improving predictive outcome, which may help reduce the reliance on subsequent animal testing in drug development. We compared two candidate human hepatic cell lines, HepaRG and C3A cells, and demonstrated differential organotypic properties. Only HepaRG cells retain differentiated morphological, phenotypic and augmented functional features for context‐specific applications, such as in early‐phase drug discovery and development, or BAL use. HepaRG cells may represent a more physiologically relevant pre‐clinical platform for CYP450 activation/inhibition studies and safety pharmacology, as well as drug–drug interaction studies.

Comparative assessment through the characterization and validation of functional and phenotypic properties of candidate human hepatic cells, for a defined operational task, is not widely reported 18. Compared with C3As, HepaRG co‐cultures exhibit a more organotypic phenotype, including evidence of hepatic polarity with strong expression of CYP3A4 (fig. 1), the major isoform involved in the metabolism of over 60% of marketed drugs 12, 13_._ Similarly, greater expression of CYP1A2, CYP2E1 and CYP3A4 genes in HepaRG cells, compared with C3As, is comparable with that of human liver biopsies, whilst HepG2 cells have also been shown to express very low CYP450 levels 16.

Analytical techniques including LC‐MS/MS and flow cytometry allow candidate *in vitro* models to be interrogated for cell type‐specific markers and drug reactive metabolite formation, allowing intrinsic (predictive) drug clearance rates to be calculated. Both turnover and the profile of HepaRG phenacetin and testosterone metabolites are significantly broader compared with C3As (figs. 3 and 4), suggesting their usefulness in predictive drug testing. Moreover, LC‐MS/MS profiling of phenacetin showed that only HepaRG cells catalysed a range of complex Phase I (oxidation, mono‐demethylation and tri‐demethylation) and Phase II (sulphonation and glucuronidation) reactions (see fig. 3B).

Many *in vitro* drug testing assays use HepG2 cells, whilst the HepG2 derivative 19, 20, C3A cells, has been utilized in tissue engineering and cell therapeutic applications, such as the extracorporeal liver assist device (ELAD) system. Although the ELAD system failed to meet expectations in clinical trials 21, it is well known that detoxification capacity and mixed function oxidase activity are very poor 22, in HepG2 cells, which require the manipulation to stimulate CYP450 activity such as transfection 23, whilst metabolic preconditioning 6 can enhance functional suitability for BALs, drug safety or pharmacological experiments. Although useful in proliferation assays, further reported disadvantages of HepG2 cells, which we show in the present study, include the lack of specific liver function (CYP450 activity/metabolism) that potentially does not have the significant toxic effects of candidate compounds under investigation.

C3A cells are, however, a useful model for context‐specific applications including predictive modelling of hepatotoxicants 24, metabolomics and tissue engineering approaches including 3D‐spheroid formation 7 and in microfluidics devices 8, which improved the phenotype and functionality. This is equally true of HepaRG cells that have been utilized in clinical applications such as BALs, hepatitis research and drug‐induced liver injury (DILI) 15, 16.

Our study supports the preferential usage of HepaRG over C3A cells in such applications 12, 13 due to their reproducibility and stability (>28 days) in culture, coupled with a significantly greater array of gene and enzymatic mixed function oxidases (fig. 2). In agreement, other reports show that these cells exhibit Phase I–III metabolism 25. Indeed, Gerets *et al*. 26 compared HepG2, HepaRG and PHHs assessing gene expression and employing real‐time impedence biosensing with various CYP450 assays containing prototypical inducers. They found HepaRG cells to be the most inducible model and metabolically more closely related to PHHs than HepG2. When comparing gene expression of Phase I, II and III mRNA between HepG2, HepaRG and PHH cells, they also found that HepG2 cells expressed the lowest mRNA values for almost every gene tested 22. Altogether, these studies suggest that HepaRG may be a useful surrogate to PHHs.

We performed flow cytometric analysis to assess and compare the expression profile of cell surface integrin receptors. Integrin‐mediated cell adhesion sites link the actin cytoskeleton with the extracellular matrix and are important modulators of cell behaviour including growth and survival, differentiation and polarity 27. Expression profiling reveals that HepaRG116 differentiated cells show markers associated with hepatocyte, biliary and epithelial cells morphologically (fig. 5). In contrast with C3A, which was derived from a hepatocellular carcinoma patient, the non‐tumorigenic HepaRG cells notably express the most abundant hepatocyte integrin α_5_ (the fibronectin receptor, α_5_β_1_), important in development and cell differentiation processes. Therefore, HepaRG cells may represent a valuable model to study hepatic cell–cell/ cell–matrix interactions.

Given that hepatic clearance of drugs depends on the activity of transport proteins located on the bile canalicular membranes, the unique properties of HepaRG polarity (fig. 1) may permit focused approaches for cholestatic drug‐based DILI studies as well as drug–drug interactions, a significant bottleneck in the drug development pipeline 15. This, together with the broad hepatic functionality of the HepaRG *in vitro* co‐culture system, also offers an opportunity to establish robust ‘disease‐in‐a‐dish’ models and offers insight into the interaction between hepatocytes and cholangiocytes, a main feature of *in vivo* hepatic organization.

One disadvantage of the HepaRG cells is currently their cost when compared with the widely available HepG2/C3A cells, which can be propagated through multiple passages. This is perhaps offset to some degree, by the stability of the HepaRG cells for a variety of time‐sensitive experiments such as acute, chronic or repeat‐dose hepatotoxicity studies, over 4 weeks, so enabling a larger number of experiments with less variability and more stability.

In conclusion, our comparative study supports human HepaRG cells as a more pertinent organotypic *in vitro* human model, compared with C3A cells, which may permit more predictive pre‐clinical screening of drugs and could potentially reduce drug attrition, DILI and animal testing, leading to lower drug development costs. Defining physiological properties of such hepatic models may inform future human liver tissue modelling strategies for a variety of pharmaceutical and therapeutic applications.

## Supporting information


**Appendix S1.** Methods.
