# Supplementary material for: Human Hepatic HepaRG Cells Maintain an Organotypic Phenotype with High Intrinsic CYP450 Activity/Metabolism and Significantly Outperform Standard HepG2/C3A Cells for Pharmaceutical and Therapeutic Applications
Source: Basic Clin Pharmacol Toxicol. 2016 Jul 15;120(1):30–7. doi: 10.1111/bcpt.12631 (PMC5225883; doi:10.1111/bcpt.12631)
Supplement: Supplementary file 1 — Appendix S1. Methods. [file BCPT-120-30-s001.docx]

**Supplementary Information**

# Human hepatic HepaRG cells maintain an organotypic phenotype with high intrinsic CYP450 activity/metabolism and significantly outperform standard HepG2/C3A cells for pharmaceutical and therapeutic applications

*Leonard J Nelson^1^, *Katie Morgan^1^, *Philipp Treskes^1^, Kay Samuel, Catherine J Henderson^2^, Claire LeBled^1^, Natalie Homer^2^, M Helen Grant^3^, Peter C Hayes^1^, John N Plevris^1^

^1^Hepatology Laboratory, University of Edinburgh, Royal Infirmary of Edinburgh, Edinburgh, United Kingdom; ^2^Mass Spectrometry Core Laboratory, Wellcome Trust Clinical Research Facility, Queen's Medical Research Institute, Edinburgh, United Kingdom; ^3^Scottish National Blood Transfusion Service, Research, Development and Innovation Directorate, Cell Therapy Group, Ellens Glen Road, Edinburgh; ^4^Department of Biomedical Engineering, University of Strathclyde, Glasgow, United Kingdom

**Methods**

**LC-MS/MS**

**Phenacetin metabolism: Liquid chromatography-mass spectrometry**

Phenacetin metabolism was assessed using an ABI Applied Biosystems 5500 QTrap Mass spectrometer and AB Sciex software, Analyst 1.5.1; and LightSight software to identify metabolites. Methods are described in detail in Supplementary Information. the sample (1µg/ml) was directly infused into an ionisation source, operated in positive electrospray ionisation mode.

The tunefile for phenacetin (molecular weight 179.2 Da) was loaded into the software package LightSight, for metabolite identification, using Phenacetin parent-product transitions. Metabolites were predicted by the software and criteria of Phase I and Phase II metabolism was monitored for each experimental injection. Samples were injected onto an ACE Excel C18 (100 x 3 mm; 2 um) fused core column, with 0.1% formic acid in water and 0.1% formic acid in methanol as the mobile phase. An Acquity UPLC autosampler was used, coupled to the QTrap 5500 triple quadrupole. The ion trap capability of the QTrap third quadrupole, enables excellent assessment of metabolites in complex mixtures.

The quantity of metabolites was assessed and reported as a percentage of the peak area of the parent molecule. Coupling the observation of phenacetin metabolites through the LightSight software, and using our previously reported LC-MS/MS method, used for the quantitative analysis of paracetamol (a metabolite of phenacetin), and its Phase I and Phase II metabolites in urine^16^, we were able to identify with confidence, analytes and their corresponding metabolites within our samples, based on mass transitions and retention times on the column.

For detection of phase 2 metabolism, a control sample of 10 µg paracetamol was used as well as samples of human urine, from subjects administered paracetamol A base line of paracetamol and its metabolites was created using a sample of human urine from a patient that had received a therapeutic dose of paracetamol. This sample of urine was used to confirm the retention times of the metabolites of paracetamol using the LC-MS method.
